# Network Topology and Undominated Assembly Processes Govern Soil Nematode Community Responses to Forest Type

**DOI:** 10.3390/microorganisms14051147

**Published:** 2026-05-19

**Authors:** Bing Yang, Zhihe Zhang, Yue Liu, Zhidi Wang, Yuanlan Sheng, Zhisong Yang

**Affiliations:** 1 Conservation of Endangered Wildlife Key Laboratory of Sichuan Province, Sichuan Academy of Giant Panda, Chengdu 610081, China; zhidi_wang@163.com (Z.W.); 18623481317@163.com (Y.S.); 2 Giant Panda National Park Chengdu Administration, Chengdu 610096, China; zhangzhh@163.com; 3 Key Laboratory of Sustainable Forest Ecosystem Management—Ministry of Education, School of Forestry, Northeast Forestry University, Harbin 150040, China; yueliu0211@163.com

**Keywords:** amplicon sequencing, bioindicator, forest soil quality, soil nematode community, community assembly, network ecology

## Abstract

Soil nematodes are integral to soil micro-food webs and serve as sensitive bioindicators of soil ecological condition. However, how forest vegetation and soil properties interact to shape nematode community assembly, network structure, and functional stability remains inadequately understood. Using 18S rRNA gene amplicon sequencing coupled with phylogenetic null modeling, we examined soil nematode communities across four forest types along a succession gradient. Although taxonomic diversity (e.g., Shannon and Pielou indices) differed significantly among forest types, network topology and stochastic assembly processes were more closely associated with community restructuring and co-occurrence patterns. This suggests that, while diversity is not irrelevant, network- and assembly-based metrics provide complementary and often more sensitive indicators of nematode community responses to forest type. Co-occurrence network analysis revealed that mixed forests fostered more complex and potentially stable networks, whereas plantations formed dense but potentially vulnerable networks. Assembly processes were not dominated by strong deterministic selection (|βNTI| ≤ 2 for most comparisons), a pattern consistent with undominated processes (e.g., ecological drift, weak environmental filtering). Dispersal limitation played a negligible role in this system. Partial Least Square Path Modeling identified spatial factors and key soil properties (e.g., pH, electrical conductivity, soil water content, and organic carbon) as primary drivers of community structure. Our findings indicate that assessing soil food web health should integrate network analysis and stochasticity metrics rather than rely solely on taxonomic diversity. For sustainable forest management, mixed-species stands are preferable to monoculture plantations, and maintaining soil physicochemical heterogeneity is critical for community stability.

## 1. Introduction

Soil food webs are fundamental conduits for nutrient transfer, linking resources, consumers, and predators to drive ecosystem functioning [1]. Within these webs, nematodes represent the most abundant multicellular animals in soil and play indispensable functional roles across multiple trophic levels [2,3]. Herbivorous nematodes facilitate the transfer of root-derived nutrients to soil food webs and stimulate secretion of secondary metabolites and compensatory growth in roots [4,5]. Bacterivorous nematodes serve as key regulators of soil nutrient cycling by modulating bacterial community composition and activity, thereby promoting nitrogen and phosphorus mineralization that sustains plant productivity [6]. Fungivores nematodes drive microbial diversity and carbon cycling in soil [7]. Predatory-omnivorous nematodes maintain the complexity of soil food webs by promoting biological interactions and accelerating material and energy turnover [4,8,9]. Through herbivory, bacterivory, fungivory, and predation, nematodes regulate microbial activity, stimulate root physiology, catalyze carbon and nutrient turnover, and maintain the complexity of belowground interactions [4,5]. Consequently, nematode communities are widely recognized as sensitive bioindicators of soil condition and ecosystem health.

In forest ecosystems, nematode communities are shaped by a hierarchy of abiotic and biotic factors. While edaphic properties such as pH and soil organic carbon (SOC) strongly influence their spatial distribution [10], vegetation exerts a primary structuring role through root-derived resources, litter chemistry, and the modification of soil microhabitats [11,12]. In particular, tree species identity can alter nematode trophic structure and community composition, creating distinct assemblages under coniferous versus broad-leaved stands [13]. However, the effects of tree diversity per se on nematode taxonomic diversity remain ambiguous, with some studies reporting minimal influence compared to species identity or local soil conditions [11,14]. This suggests that forest type, integrating species composition, stand structure, and associated soil properties, may be a more critical determinant of nematode community structure than simple metrics of tree richness.

Critically, most studies to date have focused on describing patterns in nematode diversity and composition across forest types. Far less is known about how forest types regulate the interaction networks and assembly processes that underlie these communities as key determinants of ecological stability and function. The combination of these two frameworks (network topology and stochastic assembly) moves beyond a purely taxonomic inventory to understand the underlying rules and organizational structure of the community. Co-occurrence network analysis offers a powerful tool to elucidate the complexity and stability of species interactions within the soil microbiome [15]. Recent evidence suggests that forest type can significantly alter nematode network topology; for example, mixed forests foster more modular and potentially stable networks than monocultures [16]. Simultaneously, community assembly theory provides a framework to disentangle the deterministic (e.g., environmental filtering) and stochastic (e.g., dispersal limitation, ecological drift) processes that shape community structure [17]. Beyond taxonomic diversity, recent advances in plant and soil microbiome research emphasize that the restructuring of microbial communities and their interaction networks plays a critical role in ecosystem stability and functional resilience [18]. This perspective aligns with a growing recognition that community assembly processes, particularly the balance between stochastic and deterministic forces, shape not only who is present but also how species interact. Understanding these interaction networks may therefore be essential for predicting ecosystem responses to environmental change. In the context of global change, understanding whether nematode communities assemble predictably from environmental conditions or via stochastic dynamics is essential for predicting their resilience and functional responses [19].

In the present study, we address these interconnected knowledge gaps by investigating whether forest types differentially impact the diversity, interaction networks, and assembly processes of soil nematode communities. We tested three specific hypotheses: (1) community composition and diversity will vary significantly with forest type, and these patterns will be directly correlated with key proximate soil factors (e.g., pH, SOC, soil moisture); (2) the complexity and topological characteristics of nematode co-occurrence networks will differ more profoundly across forest types than will taxonomic diversity, with mixed forests supporting more stable and integrated network structures than pure-species plantations; and (3) the assembly of soil nematode communities will be dominated by stochastic processes across all forest types, as indicated by null model analysis, reflecting the importance of dispersal and ecological drift in structuring these communities. By integrating community diversity, co-occurrence networks, and phylogenetic null modeling, this study aims to advance a mechanistic understanding of how forest management and restoration strategies influence the foundational interactions and assembly rules of soil food webs.

## 2. Materials and Methods

### 2.1. Site Description

The study was conducted in the Fengtongzhai Nature Reserve, located in Baoxing County, Sichuan Province, China, with geographical coordinates ranging from 102°48′ to 103°00′ E and from 30°19′ to 30°47′ N. This reserve covers approximately 390 km^2^ and is characterized by its rugged ridges and narrow valleys, resulting in a diverse landscape with elevations ranging from 1000 to 4896 m. The region experiences significant seasonal climatic variations, with spring lasting from April to June, summer and autumn from July to October, and winter from November to March. Meteorological data indicate that the average annual temperature ranges from 5.9 °C to 7.2 °C, with humidity levels between 79% and 83%. Precipitation is substantial, with an average annual rainfall of 730 to 1300 mm. July is the warmest month, with temperatures ranging from 15.1 °C to 16.3 °C, while January is the coldest month, with temperatures ranging from −4.0 °C to 2.7 °C.

The Fengtongzhai Nature Reserve demonstrates distinct vertical zonation of vegetation [20]. Below 1500 m in elevation, subtropical evergreen broad-leaved forests are the most prevalent, dominated by tree species such as *Cinnamomum wilsonii* (Lauraceae) and *C. longepaniculatum*. As one ascends to elevations ranging from 1500 to 2000 m, the ecological composition shifts to a combination of evergreen and deciduous broad-leaved forests. This transitional zone features key deciduous species, including *Pterocarya stenoptera* (Juglandaceae), various *Betula* spp. (Betulaceae), and multiple *Acer* spp. (Sapindaceae). At altitudes between 2000 and 2900 m, the forest ecosystem further evolves into a blend of coniferous and deciduous trees, with notable species such as *Tsuga chinensis* (Pinaceae) and *Pinus armandii* (Pinaceae), as well as the deciduous *Betula* spp. Above 2900 m, coniferous forests become dominant, and at elevations exceeding 3500 m, the environment transitions to shrubs and grasslands, illustrating the diverse plant life that thrives in response to the varying altitudes within the reserve. In this experiment, a space-for-time-substitution approach was employed to investigate the ecological succession of soil nematode community alongside the primary succession of native vegetation.

A space-for-time substitution (SFT) approach, also referred to as a pseudo-chronosequence, to infer successional trajectories of soil nematode communities across different forest types (plantation, coniferous, broadleaf, and mixed). SFT assumes that spatial variation among sites at a single time point can substitute for temporal dynamics, provided that the system is in a steady state and that key environmental drivers are consistent across space. This approach is widely used in forest ecology to study succession and soil development over decadal to centennial time scales, particularly when long-term, repeated sampling is not feasible. A recent study directly validated the SFT approach for soil nematode communities by comparing a pseudo-chronosequence with a real chronosequence over 4 years of post-mining reclamation [21]. The authors found that both approaches reached similar conclusions, demonstrating that the pseudo-chronosequence approach successfully predicts the temporal development of the nematode soil food web.

### 2.2. Soil Sampling

The sampling protocol follows the approach validated in our previous study on forest soil protistan communities [20]. Briefly, four forest types were investigated: coniferous forest, deciduous broad-leaved forest, mixed forest (comprising both deciduous broad-leaved and evergreen broad-leaved species), and coniferous plantation forest. For each forest type, six independent replicate plots (20 m × 30 m) were established. Within each plot, three subplots (5 m × 5 m) were randomly positioned at least 15 m apart. In each subplot, ten soil cores (20 cm depth, 5 cm diameter) were collected in October 2020 and homogenized into a single composite sample, representing one biological replicate. Consequently, each forest type yielded 18 biological replicates (6 plots × 3 subplots), resulting in a total of 72 samples (4 forest types × 18 replicates) for molecular analysis. After removing the forest floor layer and litter materials, soil cores were taken from the 0–20 cm soil layer. Soil samples were placed in a sterilized plastic bag, sealed, stored in an icebox, and transported to the laboratory immediately. The fresh soil samples were sieved through a 2 mm sterilized sieve to remove visible roots, rocks, and other residues. Each sample was divided into three parts. One fraction was stored at 4 °C until nematode extraction (within 1 week). Another fraction was kept at −20 °C. The third fraction was air-dried for soil physicochemical analysis.

### 2.3. Soil Physicochemical Properties Determination

SWC was determined by drying 10 g of fresh soil at 105 °C for 72 h. Soil pH was measured by a pH meter (PHSJ-3 F, Shanghai INESA Scientific Instrument Co, Ltd., Shanghai, China) and electrical conductivity (EC) was measured by a conductivity pen (SINCT-TDS3031) in the water–soil suspension formed by 5 g of soil and 25 mL of water. SOC and total nitrogen (TN) were measured using an elemental analyzer (Elementar vario E.L cube, Elementar, Langenselbold, Germany) after grinding and sieving dried soil through a 100-mesh sieve. Soil total phosphorus (TP) was digested by HClO_4_ and H_2_SO_4_ from ~0.5 g soil at 380 °C for 2 h, and then determined using an auto chemistry analyzer (SmartChem 450, AMS Alliance, Rome, Italy) via the Mo-Sb anti-spetrophotography method. Soil available phosphorus (AP) was extracted using 2.5 g soil with 50 mL of 0.5 mol/L NaHCO_3_ at pH 8.5 and determined using the auto chemistry analyzer. These determinations were performed by Sichuan Huabiaoce Testing Technology Co., Ltd. (Chengdu, China).

### 2.4. Soil Nematode Extraction and Community Analysis

As suggested, the minimum soil sample size for molecular characterization of nematode assemblages was 100 g [22]. Approximately 150 g fresh soil from each sample was used to extract soil nematodes using sugar flotation and centrifugation techniques. A fine membrane filter with a pore size of 0.22 µm was used to intercept nematodes. The obtained filter membrane was used to extract total DNA using the E.Z.N.A.^®^ Soil DNA Kit (Omega Biotek, Norcross, GA, USA) following the manufacturer’s protocols. The concentration and purity of extracted DNA were evaluated with a NanoDrop 2000 UV-VIS spectrophotometer (Thermo Scientific, Wilmington, NC, USA), and the quality of the extraction was assessed through 1% agarose gel electrophoresis. The hypervariable region V4 of eukaryotic 18S rRNA gene fragments were amplified from the extracted DNA using primers 3NDf (5′-GGCAAGTCTGGTGCCAG-3′) and 1132rmod (5′-TCCGTCAATTYCTTTAAGT-3′) [23,24] under the following conditions: 95 °C pre-denaturation for 3 min; 95 °C denaturation for 30 s, 55 °C annealing for 30 s, and 72 °C elongation for 45 s, for 35 cycles; 72 °C elongation for 10 min; and then storage at 4 °C. The PCR products were recovered, purified, and electrophoresed on a 2% agarose gel for quality control. Finally, paired-end sequencing was conducted on the Illumina platform by Personal Biotechnology Co., Ltd. (Shanghai, China). Raw reads of nematode were deposited in the National Center for Biotechnology Information (NCBI) Sequence Read Archive (SRA) database (Accession Number: PRJNA1467318).

Bioinformatic processing of raw sequencing data was conducted by Shanghai Personal Biotechnology Co., Ltd. (Shanghai, China) following their standard OTU-based analysis pipeline. Raw paired-end reads were first quality filtered using Trimmomatic (v0.39) to remove adapters and low-quality bases (Phred score < 20). Overlapping paired-end reads were merged with FLASH (v1.2.11), and sequences were dereplicated. Operational Taxonomic Units (OTUs) were clustered at a 97% similarity threshold using VSEARCH (v2.21.0) within the QIIME (v1.9) framework. Chimera removal was performed using the UCHIME algorithm against the or PR^2^ reference database. Taxonomic assignment of OTUs was carried out using a curated nematode reference dataset (NemaTaxa [20] and PR^2^ (version 4.1.4) [24] via the RDP Classifier with a confidence cutoff ≥ 80%. Although ASV-based approaches offer higher resolution [25], we retained the 97% OTU threshold for three reasons: (i) it reduces over-splitting of intragenomic 18S rRNA variants that are common in nematodes [26]; (ii) it improves the stability of co-occurrence estimates (e.g., SparCC) by reducing zero-inflation and excessive sparsity [27]; and (iii) it ensures comparability with previous forest soil nematode studies that used the same threshold [28,29]. Non-nematode and non-metazoan OTUs (e.g., fungal, plant, or protist origins) were removed to retain only nematode-derived sequences. Raw counts were normalized using variance-stabilizing transformation (DESeq2 version 1.40.2) followed by relative abundance scaling. Only taxa with mean relative abundance > 0.01% were retained. Finally, the OTU table was filtered to remove singletons and rarefied to the minimum sequencing depth across samples to standardize read counts for subsequent ecological and network analyses. All steps followed the company’s standard operating protocol for soil nematode community profiling.

### 2.5. Statistical Analysis

The Kolmogorov–Smirnov test was used for a normality test, and Levene’s test was used for the homogeneity of variance test before the post hoc test. The alpha diversity of soil nematode community was assessed using indices of observed OTU-richness, Faith’s phylogenetic diversity (PD), the Shannon–Wiener’s diversity (H′), Pielou’s evenness (*J*’) and Simpson’s dominance (λ). Differences in alpha diversity among forest types were tested using one-way ANOVAs or non-parametric tests, depending on the normality and homogeneity of the variance. If a significant effect was detected, the differences were further assessed using Tukey’s HSD test or pairwise t-test with the “Benjamini–Hochberg” method to adjust the *p*-values. Principal coordinate analysis (PCoA) was performed to determine the beta (β) diversity of soil nematode community according to the Bray–Curtis distance. Similarity percentages (SIMPER) analysis and Random Forest model were further subsequently combined and used to identify nematode genera that contributed most to the average dissimilarity between forest types. EnvFit function (“vegan” package version 2.7-2) [30] was used to determine the significance of each soil properties. The topological characteristics and complexity of co-occurrence networks derived from soil nematode communities across four forest types were compared with a structured analytical framework. Firstly, robust correlation matrices using SparCC was constructed from species abundance data for each forest type, applying consistent filtration thresholds (*p* < 0.05, *r* > 0.6) to define edges. The r > 0.6 threshold was selected based on (i) previous nematode network studies [28,29]; (ii) a permutation test (n = 1000) showing that random correlations exceeded 0.6 in only 0.5% of iterations (*p* < 0.005); and (iii) the observation that lower thresholds (*r* > 0.5) introduced > 40% more edges dominated by weak, potentially spurious correlations, while higher thresholds (r > 0.7) excluded known ecologically meaningful interactions documented in the literature. Key global topological metrics, including network size, connectance, average degree, and average path length, were calculated to describe overall network complexity and integration. Furthermore, modularity was assessed to quantify niche compartmentalization, and the small-world index (σ) should be computed to evaluate network efficiency and stability. Statistically significant differences in these metrics among forest types were tested via Kruskal–Wallis tests. Visual comparison of network graphs, complemented by centrality analyses (e.g., degree, betweenness) to identify keystone taxa, were used to elucidate how forest type influences the inferred interaction structure, niche breadth, and potential stability of the soil nematode community. Assembly processes of the soil nematode community were to discern the relative importance of deterministic and stochastic processes in soil nematode community development by calculating the beta nearest taxon index (βNTI) using “qpen” from the “iCAMP” package in R (version 4.5.2) based on high-throughput sequencing data [31], according to previous studies [32,33,34]. If |βNTI| > 2, the shift between the compositions of each community is dominated by a deterministic process; otherwise, it is dominated by a stochastic process. The group of |βNTI| > 2 was further divided into homogeneous selection, in which the shift between community compositions is smaller than expected (βNTI < −2), and variable selection (or heterogeneous selection), in which the shift between community compositions is larger than expected (βNTI > 2). Within the group of |βNTI| < 2, to further distinguish the subfractions of stochastic processes, we calculated the Bray–Curtis-based Raup–Crick metric (RCbray). We specifically adopted the following discriminative methods: if RCbray > 0.95, the stochastic process is considered as dispersal limitation; RCbray <−0.95 as homogenizing dispersal; and |RCbray| < 0.95 as undominated.

Finally, a Partial Least Squares Path Model (PLS-PM) was constructed to elucidate the potential relationships among geographic factors, soil physicochemical properties, nematode diversity, network complexity, and assembly process. Variable selection was performed using a two-step reduction procedure (VIF screening + confirmatory factor analysis with loadings > 0.60), guided by established soil food web frameworks. Over-fitting was assessed using 10-fold cross-validation (Q^2^: 0.12–0.38), bootstrap stability analysis (5000 resamples; *r* = 0.99 between original and bootstrapped coefficients), and parsimony metrics (PGFI = 0.47 > 0.4). Model fitness was evaluated using the Chi square (χ^2^), comparative fit index (CFI), goodness-of-fit index (GFI), root mean square error of approximation (RMSEA), normalized fit index (NFI), and Tucker–Lewis index (TLI). Models were considered to have a good fit when 0 ≤ χ^2^/*df* ≤ 2, as well as when *p* > 0.05. The significance level was set at *p* < 0.05 for all analyses unless otherwise stated.

## 3. Results

### 3.1. Community Composition, Shared Taxa, and Dominant Genera of Soil Nematodes

**Figure 1:**
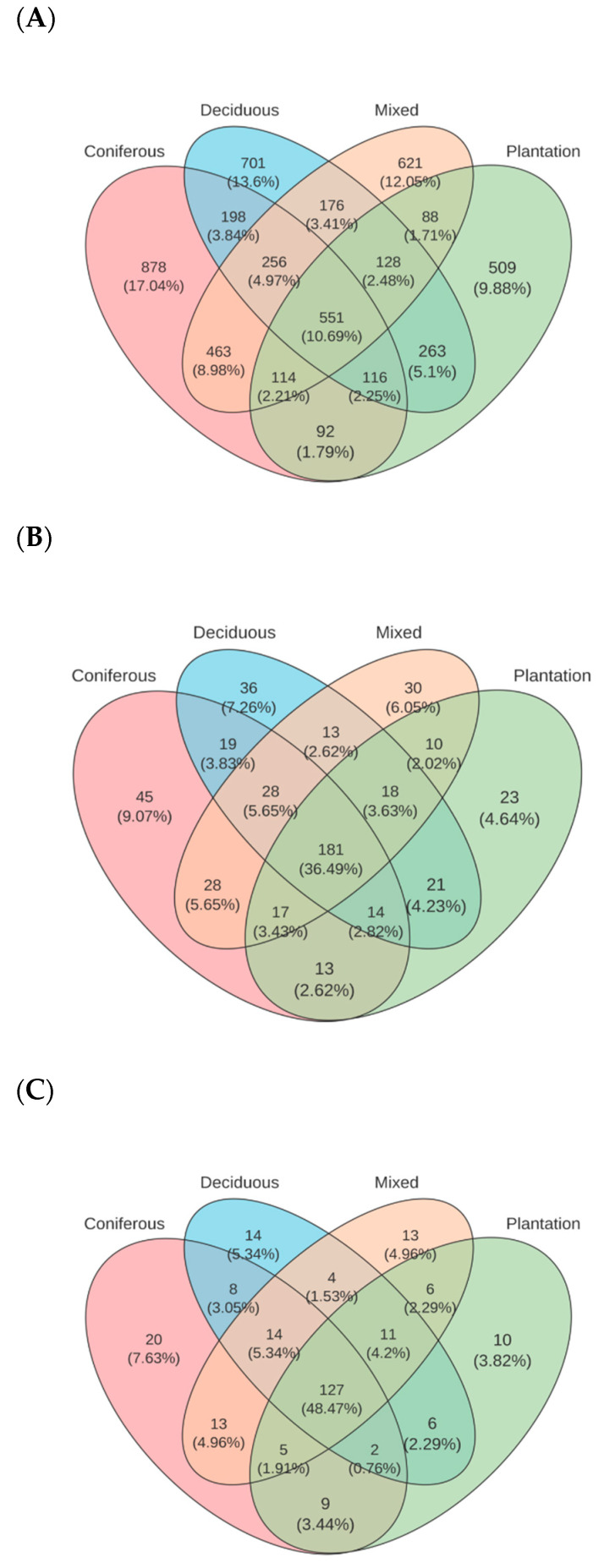
Venn diagrams of shared and unique soil nematode taxa across four forest types. Venn diagrams show the number and percentage of operational taxonomic units (OTUs) at 97% similarity (**A**), species (**B**), and genera (**C**) of soil nematodes in the topsoil (0–20 cm) of coniferous forest (Con), deciduous broad-leaved forest (Dec), natural mixed forest of deciduous and evergreen broad-leaved trees (Mix), and plantation forest (Pla). Numbers in overlapping regions indicate taxa shared between forest types; unique numbers are shown in non-overlapping regions. Percentages are calculated relative to the total number of taxa in each respective taxonomic level. Sample size: n = 18biological replicates per forest type.

The shared OTUs across the topsoil of the four forest types was 551, representing 10.69% of all OTUs (Figure 1A). Among natural forests, 256 OTUs were shared, accounting for 4.97% of the total nematode OTUs, while unique OTUs for each forest type included 878 for coniferous forests, 701 for deciduous broad-leaved forests, 621 for mixed forests, and 509 for plantation forests. At the species level, 181 shared species were observed across the four forest types, making up 36.49% of all identified species, with unique species counts of 45 for coniferous, 36 for deciduous, 30 for mixed, and 23 for plantation forests (Figure 1B). Furthermore, the shared genus count was 127, representing 48.47% of all genera, with unique genera being 20 for coniferous, 14 for deciduous, 13 for mixed, and 10 for plantation forests (Figure 1C).

**Figure 2:**
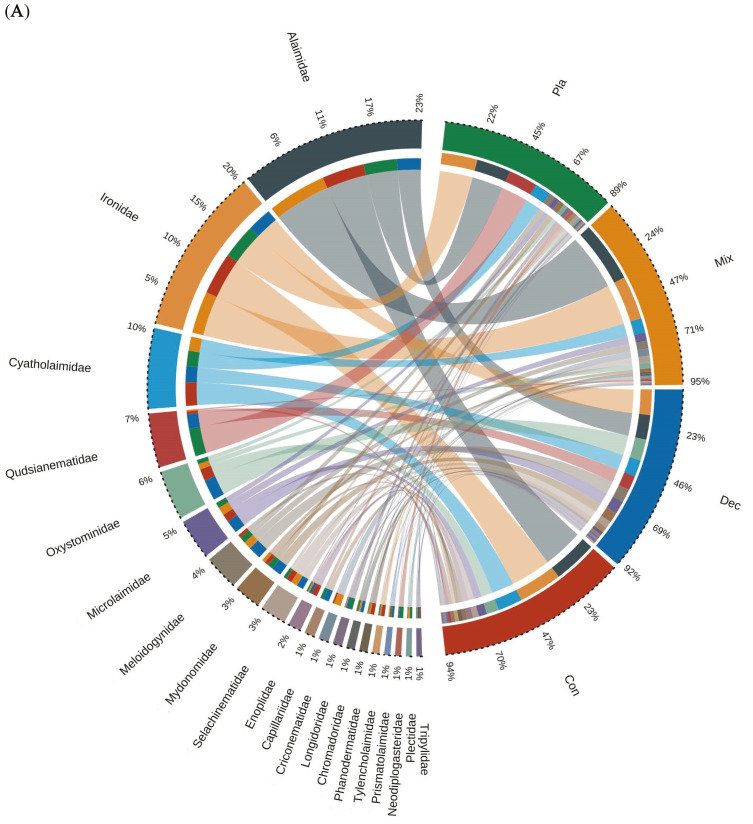

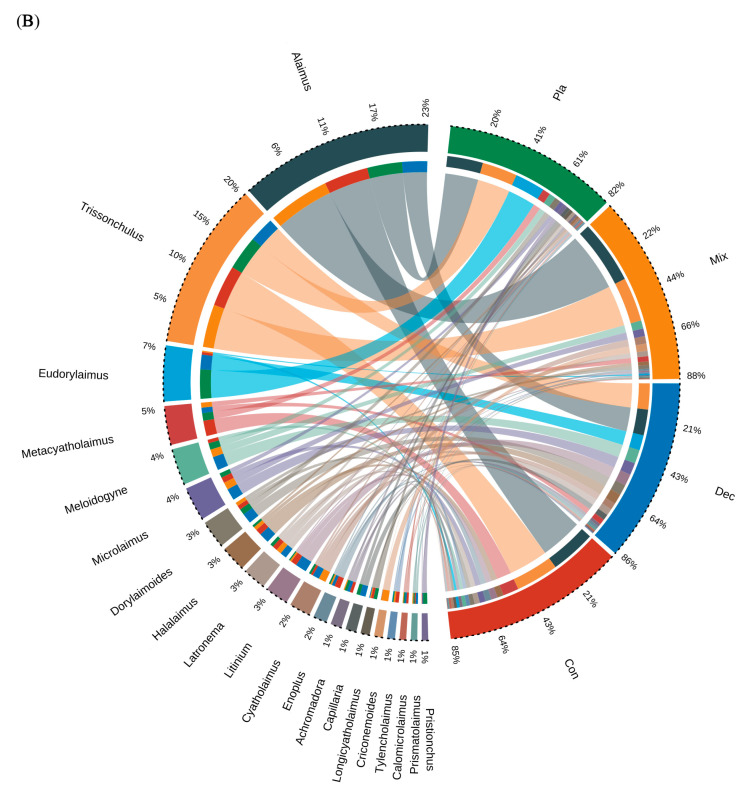

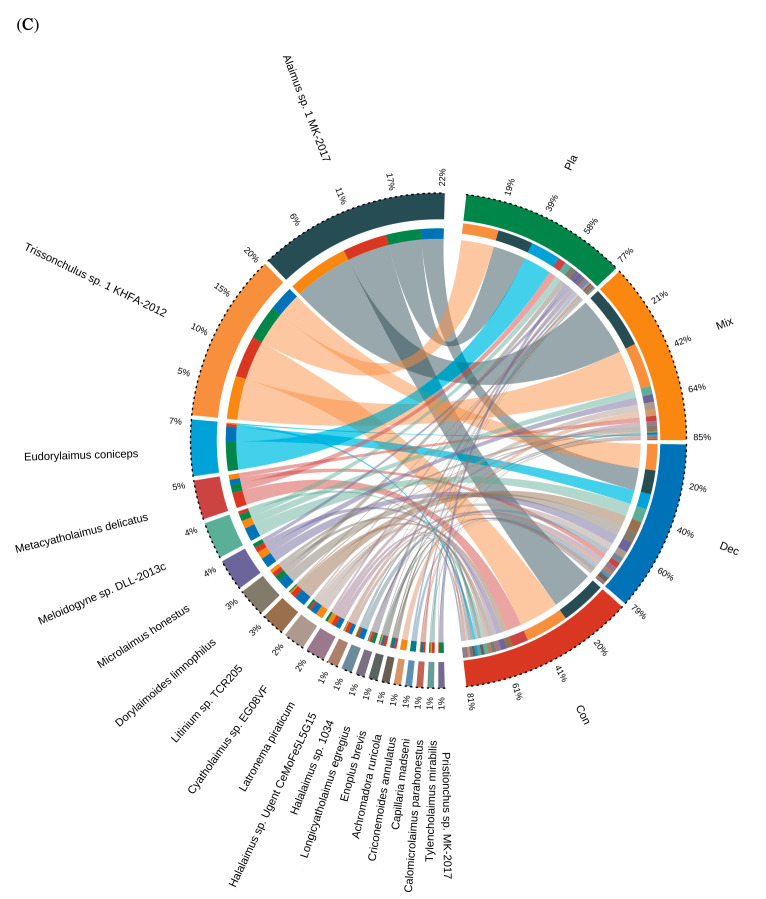
Relative abundance and interconnectivity of dominant soil nematode taxa across forest types. Chord diagrams illustrate the relative abundance distribution and inter-forest connectivity of the top 20 families (**A**), genera (**B**), and species (**C**). Each bar on the outer ring represents a forest type (colored), and the length of the bar indicates the relative abundance of the taxon within that forest type. Ribbon widths represent the proportion of the taxon’s total abundance contributed by each forest type. Only taxa with average relative abundance > 0.5% are shown.

The dominant genera in the soil nematode community, based on relative abundance, include *Acrobeloides*, *Cephalobus*, *Plectus*, *Primatolaimus*, *Dorylaimus*, *Tylenchus*, *Panagrolaimus*, and *Eudorylaimus*, collectively accounting for 76.3% of the total nematode population (Figure 2A). Correlation analysis revealed that the associations between the relative abundance of soil nematode taxa and environmental factors varied depending on nematode genera identity (Figure S1A). Across the coniferous, deciduous, mixed, and plantation forests, nine out of the ten most abundant genera were shared, with *Acrobeloides* consistently recognized as the dominant genus, exhibiting relative abundances ranging from 16.72% to 24.17%. Bacterivorous nematodes constituted the predominant functional group across forest types, comprising 52% to 60% of the total nematode abundance.

In terms of relative abundance variability, *Acrobeloides* exhibited the most significant fluctuations among the shared genera, with the highest abundance recorded in mixed forests (24.17%) and the lowest in plantation forests (16.72%), reflecting a notable difference of 7.45%. *Dorylaimus* showed the highest abundance in plantation forests (9.76%), while mixed forests had the lowest (6.89%). *Tylenchus* was most abundant in mixed forests (7.31%) but least prevalent in plantation forests (4.87%).

The unique dominant genera, *Helicotylenchus*, was exclusively found among the top ten genera in deciduous forests, with an abundance of 3.56%, while it was absent from the top ten in other forest types. Conversely, *Aphelenchus*, was unique to mixed forests, with an abundance of 2.65%. Notably, *Mononchus*, was shared only between coniferous and plantation forests, with abundances of 2.87% and 3.94%, respectively.

At the functional guild level, bacterivores were the dominant guild across all forest types, with their relative abundance ranging from 34.51% to 46.23% of the soil nematodes, followed by omnivores (21.96% to 30.51%) and predators (14.54% to 27.11%, Figure 2B). Fungivores accounted for the lowest proportion of nematodes, ranging from 0.72% to 2.39%. Further statistical analysis indicated no significant differences in most guilds among the four forest types (*p* > 0.05), with the exception of parasites (*F* = 4.04, *p* = 0.011). Specifically, deciduous forests exhibited significantly higher percentages of parasites compared to coniferous forests (*p* = 0.026) and mixed forests (*p* = 0.033).

### 3.2. Diversity Pattern and Primary Environmental Factors

**Figure 3:**
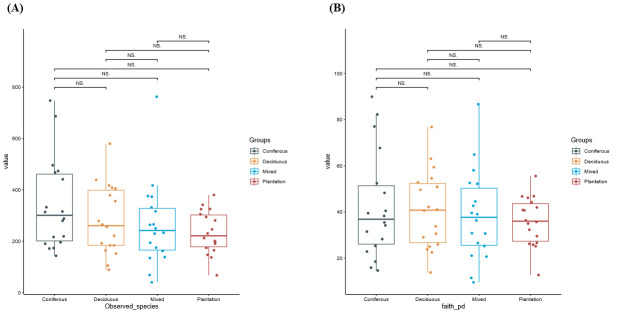

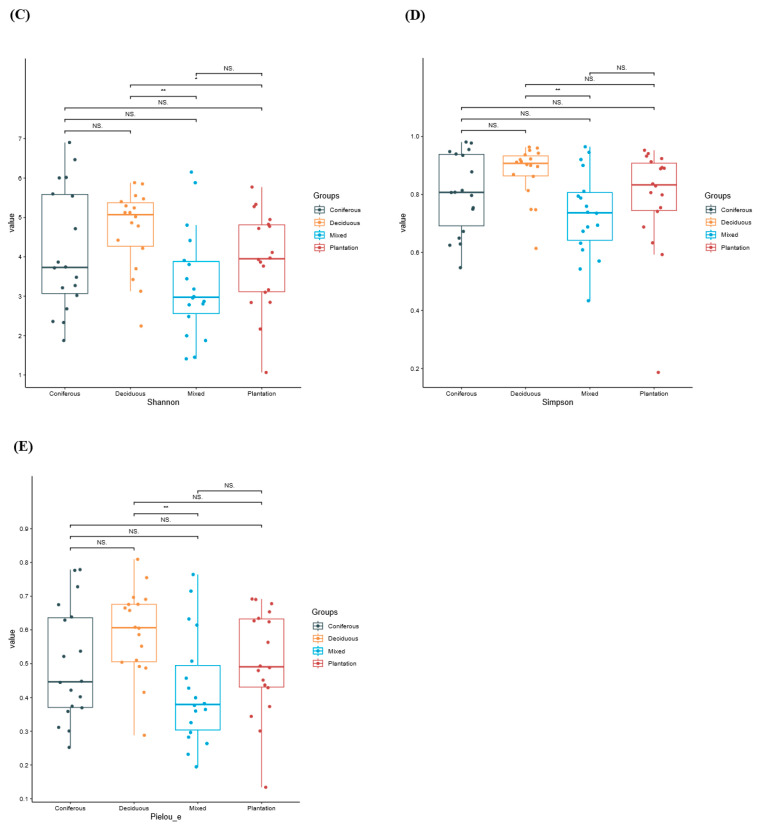
Alpha diversity of soil nematode communities across four forest types. Box plots of observed OTU richness (**A**), Faith’s phylogenetic diversity (PD) index (**B**), Shannon–Wiener’s diversity index (*H*′) (**C**), Simpson’s dominance index (*λ*) (**D**), and Pielou’s evenness index (*J*′) (**E**). For each box: center line = median; box limits = interquartile range (IQR); whiskers extend to 1.5 × IQR; points beyond whiskers are outliers. Surrounding shaded areas show kernel density distributions. n = 18 per forest type. NS indicates no significant difference between groups (*p* ≥ 0.05); * indicates *p* < 0.05; ** indicates *p* < 0.01. Significance levels are based on pairwise comparisons following the Kruskal-Wallis test, with Benjamini–Hochberg correction.

**Figure 4:**
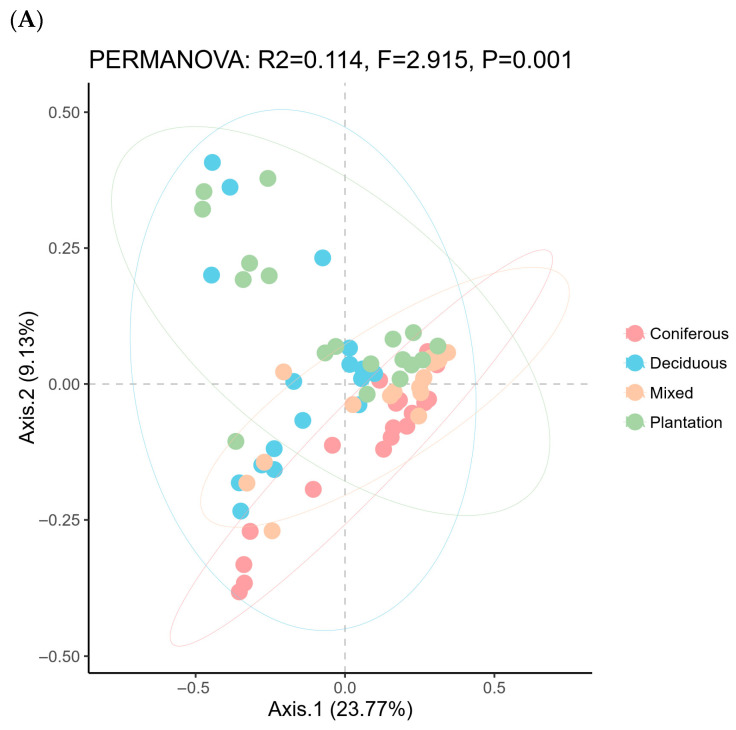

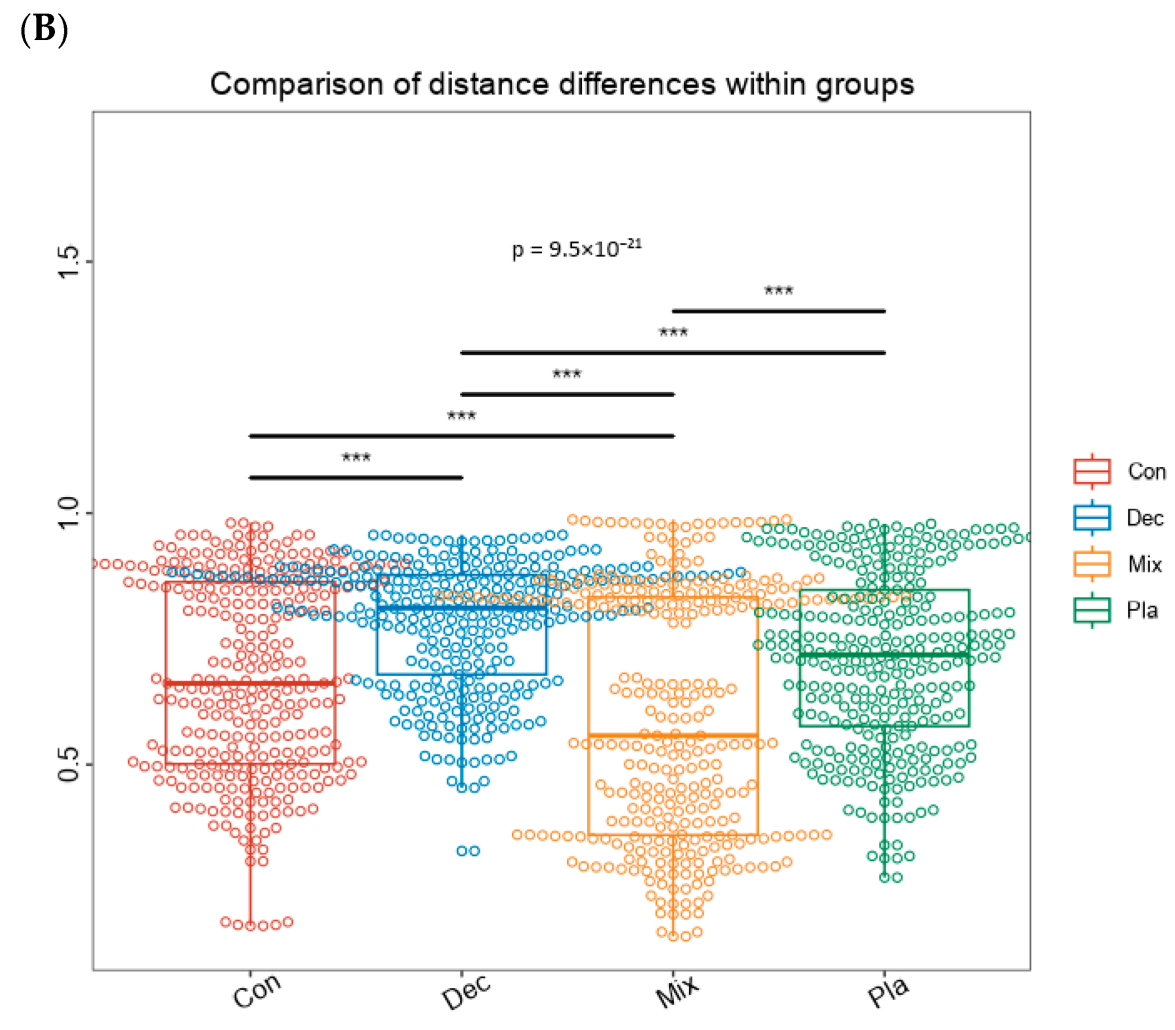
Beta diversity ordination of soil nematode communities at the topsoil layer of forest soils. (**A**) Principal coordinate analysis (PCoA) plot based on Bray–Curtis dissimilarities calculated from OTU relative abundances (n = 72 samples, 4 forest types × 18 replicates). Ellipses represent 95% confidence intervals around group centroids. Axes 1 and 2 explain 23.77% and 9.13% of total variance, respectively. Forest types explain a significant portion of community dissimilarity (PERMANOVA, *F* = 2.915, *r*^2^ = 0.114, *p* = 0.001, 999 permutations). Point shapes and colors correspond to forest types as indicated in the legend. (**B**) Within-group distance differences among soil nematode communities of coniferous (Con), deciduous (Dec), mixed (Mix), and plantation (Pla) forests based on Kruskal-Wallis test. ***, *p* < 0.001.

The difference in alpha diversity indices of soil nematode community across forest types varied greatly with diversity index identity. Observed OTU richness (Kruskal–Wallis test: *χ*^2^ = 10.73, *df* = 3, *p* = 0.013, Figure 3A) and Faith’s PD indices (one-way ANOVA: *F*= 0.985, *df* = 3, *p* = 0.408, Figure 3B) of soil nematode communities were comparable, whereas noticeable differences in Shannon–Wiener’s diversity index (*χ*^2^ = 12.45, *df* = 3, *p* = 0.006), Simpson’s dominance index (*χ*^2^ = 10.86, *df* = 3, *p* = 0.0125), and Pielou’s evenness index (*χ^2^* = 9.52., *df =* 3, *p* = 0.0231) were observed between forest types (Figure 3C–E). Specifically, the Shannon–Wiener’s diversity index, Simpson’s dominance index, and Pielou’s evenness index of soil nematode community at the topsoil of deciduous forests were significantly higher than those of mixed forests (Benjamini–Hochberg test, *p* < 0.05). Correlation analysis revealed that observed species were positively correlated to SOC and AP; Shannon–Wiener’s diversity index was positively correlated to SOC, TN, AP, EC, and longitude; and Faith’s PD index was positively correlated to SOC, TN, AN, and AP (Figure S1A). The PCoA biplot revealed the first two significant axes explaining 32.90% of total variance of soil nematode community dissimilarity (Figure 4A, PCoA1: 23.77%; PCoA 2: 9.13%). Monte Carlo test revealed there were significant differences in composition of soil nematode communities across distinct forest types (Table 1; Figure 4B). Results of EnvFit showed that altitude, latitude, pH, EC, SOC, and SWC were important factors shaping soil nematode community composition (Table 2).

### 3.3. Co-Occurrence Networks

**Figure 5:**
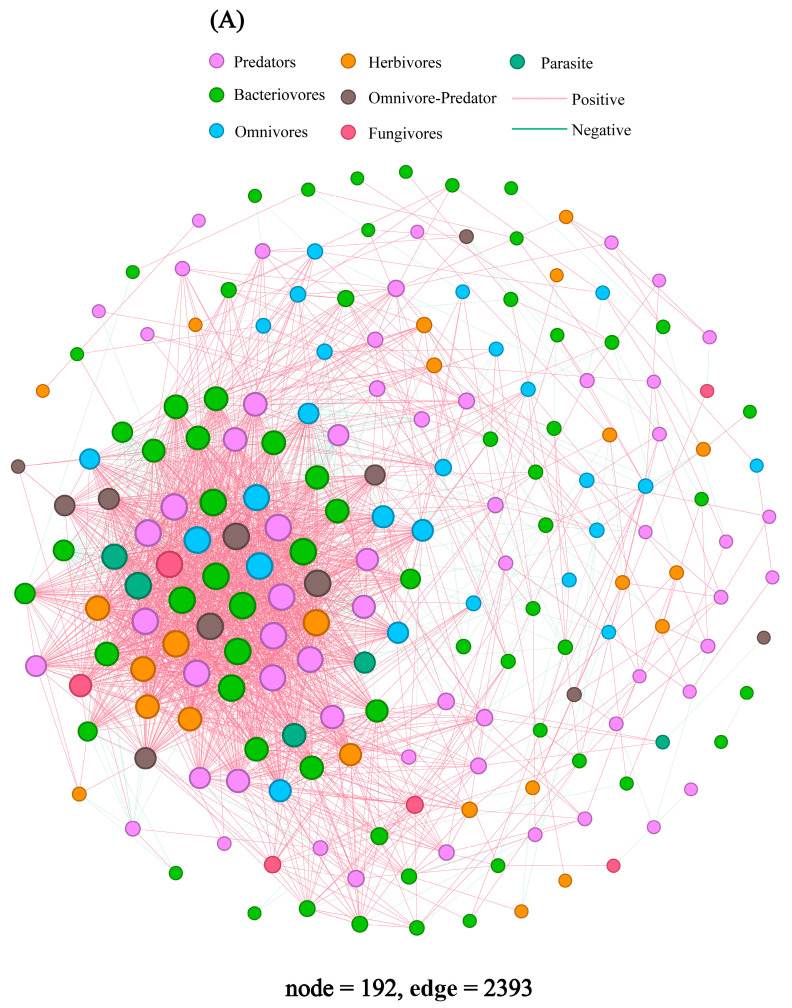

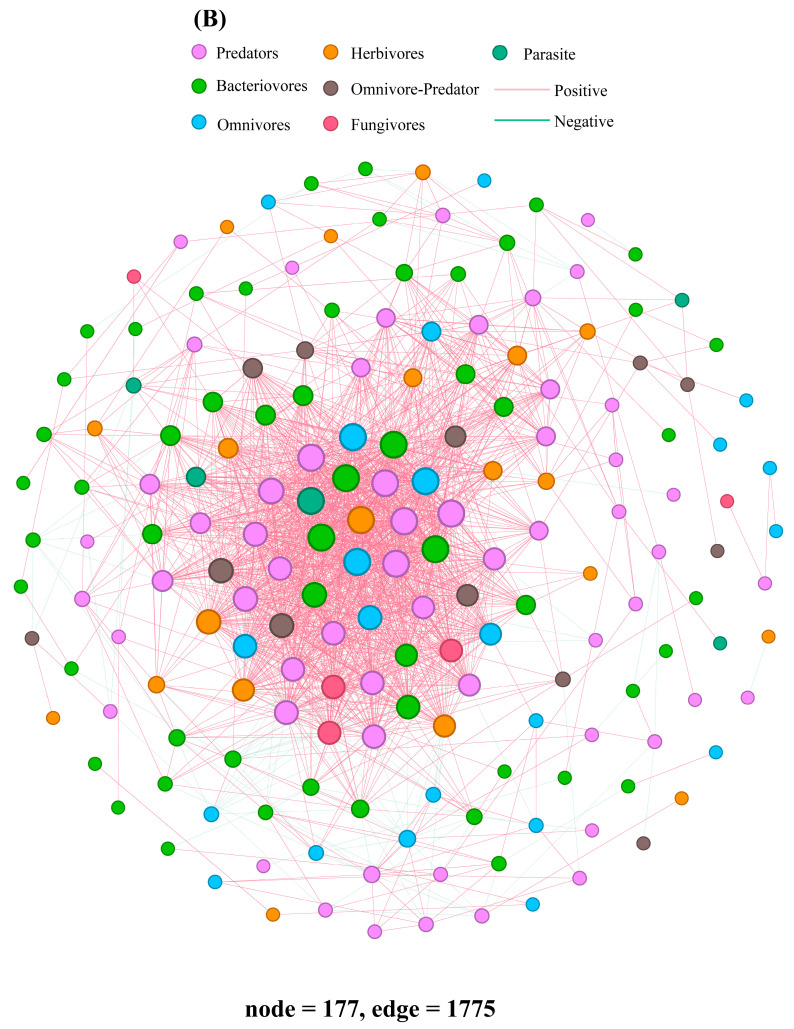

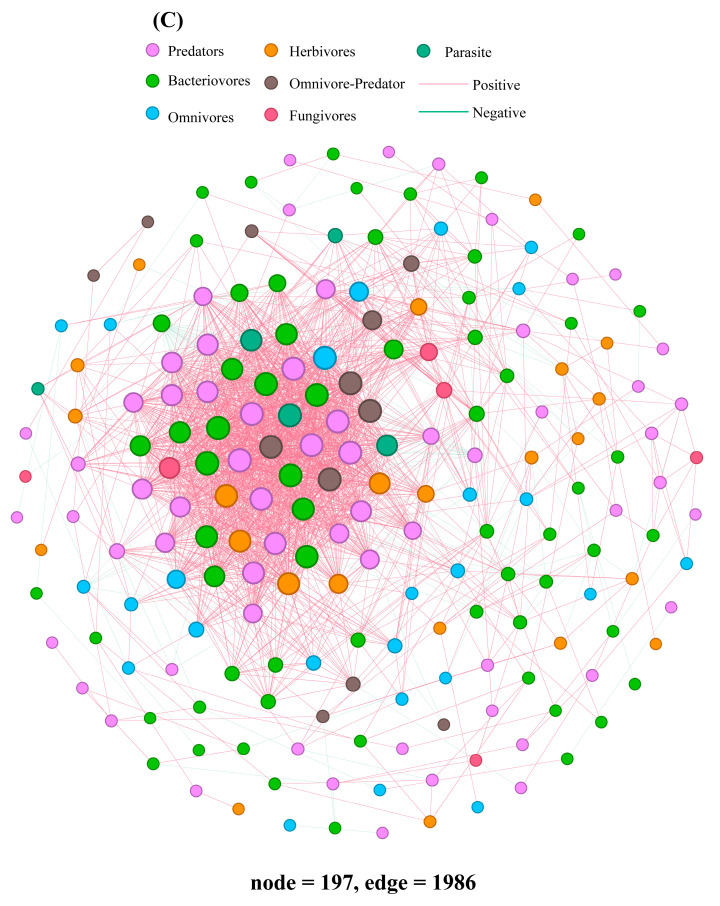

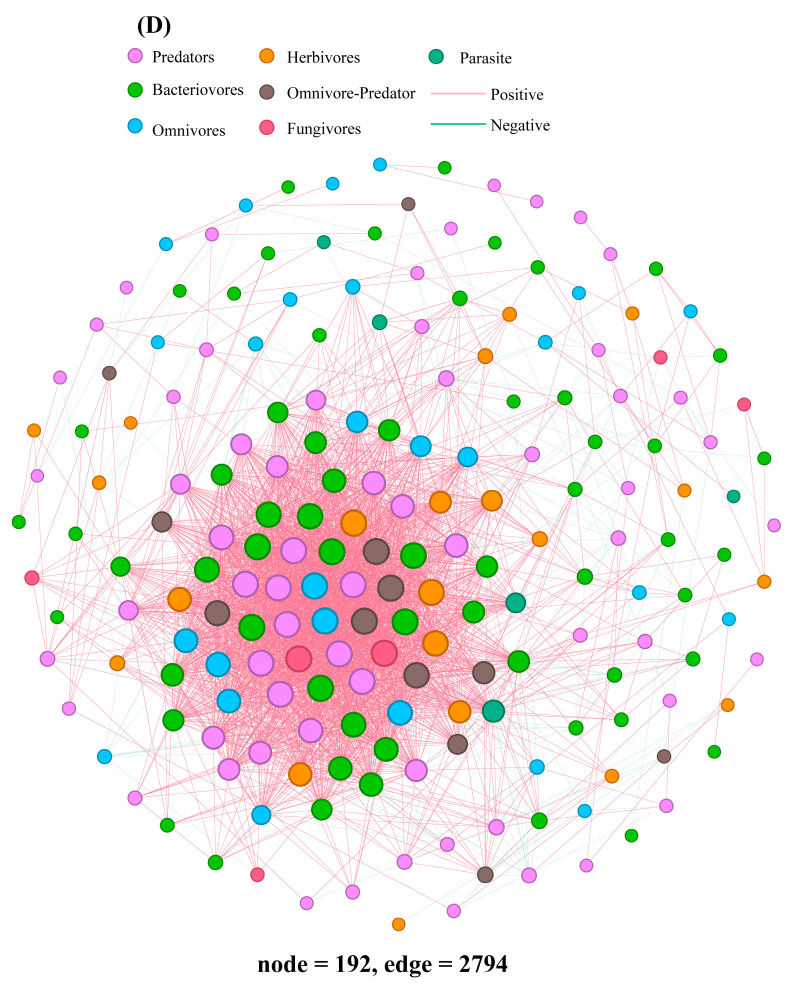
Co-occurrence networks of soil nematode OTUs in the topsoil of four forest types: coniferous forest (**A**), deciduous broad-leaved forest (**B**), mixed forest (**C**), and plantation forest (**D**). Each node represents an OTU, and node size is proportional to its degree (number of connections). Edges indicate strong and significant Spearman correlations (|ρ| > 0.6, Benjamini–Hochberg adjusted *p* < 0.05). Red edges = positive correlation; blue edges = negative correlation. Edge width scales with correlation strength. Networks were constructed using SparCC with 100 bootstrap iterations. Only OTUs present in ≥3 samples with relative abundance > 0.01% were included. Global network properties (e.g., modularity, clustering coefficient, average path length) are compared in Figure S2.

The co-occurrence networks of the soil nematode community in the topsoil of plantations and coniferous forests exhibited greater density (Figure 5). Specifically, the networks comprised 192 nodes and 2393 edges in coniferous forests (Figure 5A), 177 nodes and 1775 edges in deciduous forests (Figure 5B), 197 nodes and 1986 edges in mixed forests (Figure 5C), and 192 nodes and 2794 edges in plantations (Figure 5D).

Regarding the topological attributes of the co-occurrence networks, mixed forest demonstrated significantly higher values across multiple metrics, including clustering coefficient (Kruskal–Wallis test: *χ^2^* = 12.34, *df* = 3, *p* = 0.006), average path length (*χ^2^* = 18.95, *df* = 3, *p* = 0.010), and modularity (*χ^2^* = 28.76, *df* = 3, *p* = 0.001), compared to the coniferous, deciduous, and plantation forests (Figure S2). Conversely, the coniferous and plantation forests generally presented lower metric values.

Regarding the topological attributes of nodes within the co-occurrence networks, the differences in four examined metrics, including degree (Kruskal–Wallis test: *χ^2^* = 22.3, *df* = 3, *p* < 0.001), betweenness (*χ^2^* = 14.8, *df* = 3, *p* = 0.002), closeness (*χ^2^* = 9.7, *df* = 3, *p* = 0.021), and eigenvector centrality (*χ*^2^ = 17.5, *p* < 0.001) across the four forest types were statistically significant (Figure S3). Specifically, coniferous and deciduous forests exhibited higher values in degree and closeness, while deciduous forests demonstrated elevated betweenness. In contrast, mixed forest exhibited lower values across several metrics, and the plantation displayed moderate to higher values.

### 3.4. Phylogenetic Community Assembly

**Figure 6:**
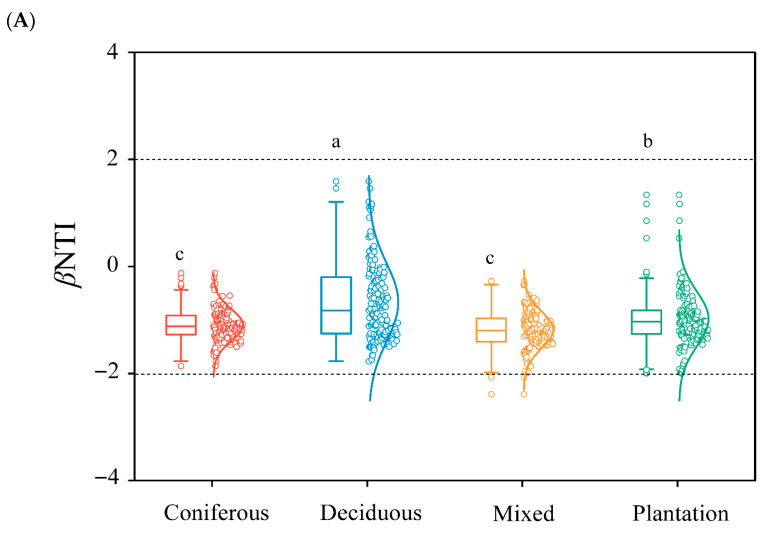

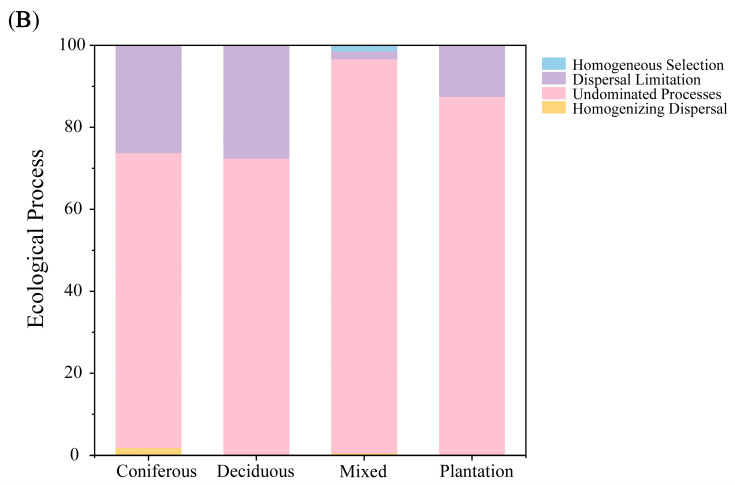

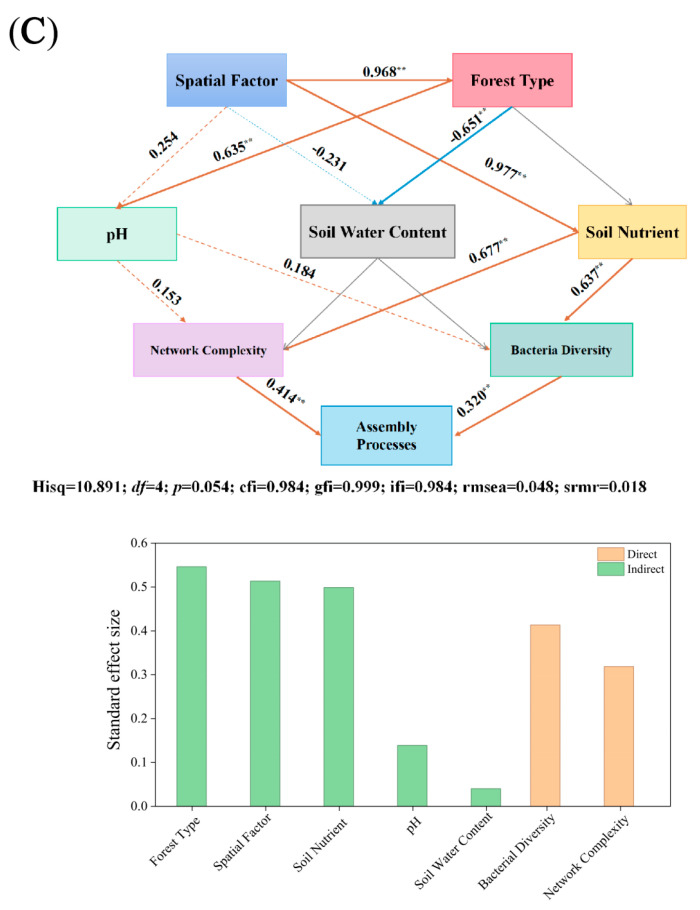
Community assembly processes of soil nematodes and their environmental drivers. (**A**) Distribution of the beta-nearest taxon index (βNTI) for pairwise comparisons within and among forest types. Horizontal dashed lines at βNTI = ±2 separate deterministic (|βNTI| > 2) from stochastic (|βNTI| ≤ 2) processes. Different letters (a, b, c) indicate statistically significant differences among groups or paths at *p* < 0.05. (**B**) Relative contributions of deterministic (homogeneous selection, variable selection) and stochastic (dispersal limitation, homogenizing dispersal, undominated = drift) processes to community assembly, quantified using the Raup–Crick metric (RCbray) for pairs with |βNTI| < 2. (**C**) Partial Least Squares Path Modeling (PLS–PM) showing direct and indirect effects of environmental factors on nematode community assembly. Network complexity is the first principal component (PC1) of clustering coefficient, modularity, edge density, average dissimilarity, average path length, and natural connectivity. Diversity = PC1 of observed OTU richness, Simpson, Pielou, and Shannon indices. Community assembly is represented by βNTI. Soil nutrient status = PC1 of soil organic carbon, total nitrogen, total phosphorus, available nitrogen, and available phosphorus. pH is measured directly. Geography = PC1 of altitude, latitude, and longitude. Orange arrows = positive effect; blue arrows = negative effect; gray = non-significant. Solid lines indicate statistically significant paths (typically *p* < 0.05), meaning the corresponding coefficients are unlikely to be zero and represent reliable relationships among variables, whereas dashed lines indicate non-significant paths (*p* ≥ 0.05), meaning the coefficients do not differ significantly from zero and should be interpreted with caution.Arrow width is proportional to standardized path coefficient. *R*^2^ values indicate variance explained for endogenous variables. Significance levels: **, 0.05 < *p* < 0.01. Model fit: χ^2^/df = 1.52, CFI = 0.96, GFI = 0.92, RMSEA = 0.07, NFI = 0.93, TLI = 0.94.

As shown in Figure 6A, most βNTI values of soil nematode communities were between −2 and 2, and the βNTI values of soil nematode communities in deciduous forests were significantly lower than those of in coniferous and plantation forests. The assembly of soil nematode communities across all four forest types was predominantly shaped by undominated processes (i.e., |βNTI| ≤ 2 and |RCbray| < 0.95), which include ecological drift and weak environmental filtering. Dispersal limitation (RCbray > 0.95) and homogenizing dispersal (RCbray < −0.95) contributed minimally or not at all in this system (Figure 6B). In contrast, homogenizing dispersal plays a minimal and consistent role in all types, while dispersal limitation appears to have no measurable influence. PLS-SEM model (*χ^2^/df* = 1.52, *CFI* = 0.96, *GFI* = 0.92, *RMSEA* = 0.07, *NFI* = 0.93, *TLI* = 0.94) suggested that forest type exerts a strong direct influence on soil properties and biotic attributes, thereby indirectly shaping the nematode assembly process, with a standardized total effect (STE) of 0.55, while soil nutrient status contributes comparably (STE = 0.51) (Figure 6C). Specifically, nematode diversity (STE = 0.41) and ecological network complexity (STE = 0.32) exert significant direct positive effects on nematode community assembly. Moreover, soil nutrients and pH influence assembly indirectly, primarily by modulating nematode diversity and network complexity. Soil nutrients show a substantial indirect effect (STE = 0.51), whereas pH exerts a modest but statistically discernible indirect effect (STE = 0.14). Collectively, structural equation modeling reveals that nematode network complexity is the strongest single driver of nematode assembly, surpassing all other measured abiotic and biotic factors in terms of both direct effect magnitude and overall contribution to explained variance.

## 4. Discussion

Our study demonstrates that forest type influences soil nematode communities through a hierarchy of mechanisms. While core guild functions are maintained, taxonomic composition and evenness-based diversity are predictably shaped by abiotic filters like SWC and pH. More profoundly, forest type reconfigures interaction network topology, with mixed forests fostering more modular and potentially stable architectures. Overarching these patterns is a community assembly regime dominated by undominated processes (including stochasticity), which may be a general feature of these highly diverse, mobile microfauna in heterogeneous soil environments. Therefore, the response of the soil nematode community to forest type is not merely a change in “who is there” (diversity/composition) but, more critically, a restructuring of “how they are organized and assembled” (network topology and assembly rules).

### 4.1. Functional Resilience and Trophic Differentiation Across Forest Types

Despite significant differences in the relative abundance of taxa across forest types (Figure 1 and Figure 2A), the overall functional guild structure of the nematode community remained relatively consistent. This finding partly aligns with the proposal that taxonomic compositions of soil nematode communities in the coniferous forest, deciduous broad-leaved forest, evergreen and deciduous broad-leaved mixed forest were similar [35]. This suggests a core functional resilience, potentially underpinned by functional redundancy among diverse taxa, allowing essential ecosystem processes to be maintained across different forest environments. Regarding the stable community composition of nematode between natural forests and plantations, a reasonable explanation is the retention effects of historical vegetation. Lower trophic level animals such as herbivores and bacterivores are more responsive to changes in plant species diversity and composition than organisms at higher trophic levels [36]. For instance, a return to nematode diversity is not reached even after 20 years following the removal of trees from the afforested prairie in Canada [37]. However, this broad stability masked important forest-type-specific functional differentiations. Most notably, plantations exhibited a distinct guild signature characterized by the highest proportion of predators and the lowest proportion of bacterivores. The co-occurrence patterns suggest a greater relative abundance of higher trophic levels in coniferous forests, which may indicate a potential shift in food web structure. In contrast, the higher abundance of bacterivorous and fungivorous genera in mixed forests (Figure 2A) points to a more robust and complex detrital pathway, supporting greater nutrient mineralization potential.

In terms of overall community characteristics, mixed forests had the highest abundances of bacterivorous and fungivorous nematode genera, with core genera showing significant dominance and the highest ecosystem complexity. In contrast, plantation forests exhibited the highest abundance of predatory genera and the lowest of bacterivorous genera, tending to form simplified predator–prey community relationships. Deciduous forests were the only type harboring the herbivorous dominant genus (*Helicotylenchus*), a phenomenon associated with the diversity of herbaceous vegetation. Coniferous forests displayed the smallest fluctuation in the abundance of core genera, demonstrating strong community stability. Consistent with these findings, herbivorous nematodes dominated cedar plantations, while bacterivorous and fungivorous nematodes prevailed in broad-leaved forests [38]. Bacterivorous nematodes are key engineers of nutrient cycling and soil health, acting as the essential link between bacteria and the larger fauna and plant roots [39], fungivorous nematodes drive microbial diversity and carbon cycling in soil [7]. Regarding *Dorylaimus*, the highest abundance was recorded in plantation forests and the lowest in mixed forests, indicating a higher dominance of predatory nematodes in plantation ecosystems. *Tylenchus* was most abundant in mixed forests and least prevalent in plantation forests. This pattern may be attributed to enhanced fungal activity during litter decomposition processes in mixed forest habitats, as previous studies have shown that most species of the genus *Tylenchus* feed on fungal hyphae, and their abundance is positively correlated with fungal activity [40]. These differences highlight the conservation value of old-growth forests as biodiversity refuges. The unique presence of dominant genera like *Helicotylenchus* (herbivore) in deciduous forests and *Aphelenchus* (fungivore) in mixed forests demonstrate that forest type acts as a strong integrative filter, shaping communities through a combination of microclimate, resource quality, and root chemistry.

The finding that parasitic nematodes were significantly more abundant in deciduous forests aligns with their role as sensitive bioindicators. Their population dynamics are tightly linked to host plant availability and condition, making their prevalence a potential signal of specific host-mediated interactions and overall ecosystem vitality within this forest type. This pattern is consistent with the role of soil nematodes as sensitive bioindicators of ecosystem status [41].

### 4.2. Community Diversity and Its Drivers

Alpha diversity patterns were metric dependent. While observed OTU richness and Faith’s PD were comparable across forests, indices incorporating evenness (Shannon–Wiener’s diversity index, Pielou’s evenness index) revealed significant differences, with deciduous forests consistently scoring highest (Figure 3C–E). This indicates that while the taxonomic breadth (richness) of nematode communities may be similar, the distribution of individuals among species is more equitable in deciduous systems. This pattern reinforces the notion that diversity indices reflecting community structure, rather than simple richness, are more sensitive indicators of forest-type influence. Additionally, the differences in diversity indices vary with climatic zones. In the temperate forest, Shannon–Wiener’s diversity index, Simpson’s dominance index, and Pielou’s evenness index in coniferous forests were lower than that of broad-leaved forests [42]. In the primary tropical lowland rainforest, Shannon–Wiener’s diversity index and Pielou’s evenness index in coniferous forests were lower than that of broadleaf forests, whereas the opposite was true was for Simpson’s dominance index [13].

The distinct community compositions (Table 1; Figure 4) also demonstrate that forest type acts as a strong integrative filter, shaping communities through a combination of microclimate, resource quality, and root chemistry. Our findings align with previous studies suggesting significant differences in nematode community composition between the broad-leaved and coniferous forests [13,43], as well as between natural forest and plantation [44,45,46,47]. Additionally, the homogeneity of soil nematode community, as evidenced by the decrease in Shannon–Wiener indices and the increase in Simpson indices, in plantation is higher than that of natural forest [45].

The primary environmental driver of community composition (β-diversity) was pH, EC, SWC, and SOC, as identified by EnvFit analysis (Table 2). The soil pH of coniferous forests is lower than that of broad-leaved forests, a phenomenon also observed in Tianshan mountain [48]. As suggested, the taxonomic diversity of nematodes and the relative abundance of bacterivores are negatively correlated with soil pH [49]. Our findings also highlight SWC as a primary driver of soil nematode community variation, consistent with its role in modulating soil nematode activity in soil ecosystems [49]. The antagonistic effects of SWC and EC suggest that water availability and nutrient retention capacity independently shape community assembly. We also observed distinct contribution of edaphic factors (Table 2) in shaping soil nematode community. Numerous studies have demonstrated that the driving factors of soil nematode community vary with ecosystems. In subalpine forests, soil nematode diversity was influenced by soil nitrogen availability [43] and SWC [50]. In subalpine coniferous forests in Wanglang, soil nematodes preferred habitats at high elevations with lower soil pH and higher SWC and AN [51]. In temperate forests, the soil C:N ratio, microbial biomass carbon, and pH were important factors affecting soil nematode communities at Changbai Mountain, China [42], whereas microhabitat and soil condition were determinants in central Japan [52]. In boreal forests in cold temperate zone of China, soil bulk density, SWC, dissolved organic carbon, temperature, pH, nitrate nitrogen, and TP were significant soil variables driving the composition of nematode community [53]. In cold-temperate montane forests, soil pH and soluble organic nitrogen were key factors shaping soil nematode community [54]. In subtropical forests, soil available nitrogen and phosphorus were key factors influencing soil nematode abundance and diversity [55]. In another subtropical forests, soil nutrient and plant diversity were found to be the shaping factor of soil nematode community [45]. Soil pH, SOC, TN, and C/N ratio significantly influenced the soil nematode community structure [56].

Our findings also demonstrated the linkages between geographic factors and soil nematode community (Table 2; Figure 6 and Figure S1). This coincides with the proposed effects of geographic factors on soil nematodes [57]. To date, the structure and diversity of soil nematodes along altitudinal gradients are still unclear, which prevents understanding and comparing the diversity of soil nematodes at different spatial scales [54]. Some studies report there is significant effect of elevation gradient on soil nematode community [10,53,54,58,59,60,61,62,63], whereas others indicate no significant effect of elevation gradient on soil nematode community [64,65].

### 4.3. Co-Occurrence Network Complexity and Stability

Co-occurrence network analysis revealed fundamental differences in interaction potential that were more pronounced than taxonomic diversity patterns. While plantations and coniferous forests formed networks with the highest number of edges (i.e., potential interactions), suggesting high complexity (Figure 5A,D), mixed forests formed networks with superior topological attributes indicative of stability (Figure S2). Specifically, mixed forest networks exhibited significantly higher modularity, clustering coefficient, and average path length. High modularity suggests the presence of distinct ecological modules, which can enhance functional stability by containing perturbations within modules [66]. However, we caution that Spearman correlations reflect co-occurrence patterns only, not verified ecological interactions. Our network results are exploratory and hypothesis-generating, and they should be interpreted as potential associations rather than definitive evidence of species interactions. This network architecture aligns with the more complex habitat and resource diversity offered by mixed-species stands. Conversely, the dense but less modular networks in plantations may be more vulnerable to cascading effects following disturbance, despite their apparent connectivity.

### 4.4. Community Assembly Processes

A central finding of this study is that the assembly of soil nematode communities across four forest types was shaped predominantly by undominated processes, as evidenced by the majority of βNTI values falling between −2 and +2 (Figure 6A). This result robustly supports our third hypothesis. Contrary to the expectation that contrasting forest types would impose strong deterministic filtering, our results consistently showed a lack of strong deterministic selection across forest types (|βNTI| < 2). These findings advance current theory in two key respects. Firstly, it challenges the prevailing assumption that environmental heterogeneity, such as differences in litter quality, soil pH, and microclimate among forest types, necessarily increases the role of deterministic selection (niche filtering) in community assembly. Our results suggest that undominated processes (e.g., dispersal limitation, ecological drift, or historical contingency) can override deterministic forces even in ecologically distinct habitats. Additionally, our findings align with an emerging perspective that soil food webs may be inherently more subject to undominated processes than above-ground communities, potentially due to high dispersal rates, cryptic spatial structure, or the buffering effect of the soil matrix on environmental extremes.

The prevalence of undominated processes can be attributed to several factors inherent to nematode ecology in forest soils: high dispersal potential via water and soil movement; fine-scale heterogeneity of soil microhabitats creating a mosaic of neutral niches; and, potentially, historical colonization legacies. This finding is consistent with a growing literature emphasizing the role of undominated processes in soil communities and suggests that, at the spatial and taxonomic resolution of our study, random demographic events and dispersal limitation are primary forces structuring these communities. The increased influence of undominated processes in mixed stands may further contribute to their higher diversity and network stability by allowing for greater historical contingency and species coexistence.

The relative importance of deterministic versus stochastic processes in structuring soil nematode communities remains a subject of active research. While some studies emphasize the role of deterministic, niche-based processes [67,68,69,70], a growing body of evidence, including our findings, suggests that stochastic processes often dominate soil nematode community assembly [32,33,61,71]. This prevalence can be attributed to several key factors related to nematode biology and their environment. Firstly, soil nematodes are highly mobile, capable of dispersing over considerable distances via soil movement, water, and on plant roots. This mobility facilitates the random colonization of available niches, leading to community structures that are not solely dictated by environmental filters. Secondly, the inherent heterogeneity of soil, with its variations in microhabitats, moisture, texture, and organic matter, creates a mosaic of micro-niches. This patchiness allows diverse nematode species to establish through random colonization events, irrespective of strong deterministic filters. Furthermore, in stable environments with conditions suitable for tree growth and weak regional environmental gradients, the influence of undominated processes is often enhanced [32].

The dominance of undominated processes in soil nematode assembly has significant ecological implications. The increased relative contributions of stochastic processes could prompt the survival of nematode species with poor environmental adaptability and lead to the increases in nematode diversity and community complexity [72]. This underscores the importance of both random demographic events and historical context in shaping the intricate communities within the soil. Taken together, our results suggest that undominated processes can govern soil nematode community assembly even across distinct forest types—a context where deterministic filtering is often assumed to dominate. We therefore advocate for a more nuanced theoretical framework that accounts for the interaction between niche-based and neutral processes, rather than assuming environmental heterogeneity automatically implies deterministic assembly. Further studies are needed to explicitly test whether the dominance of undominated processes in soil nematode communities is a general phenomenon across broader spatial and temporal scales, or a context-dependent outcome of specific succession or disturbance histories.

### 4.5. Limitations and Implications

This study is limited by the use of data obtained from a single sampling time point, which may not provide a comprehensive understanding of soil nematode distribution [73]. Previous studies reported distinct seasonal pattern of soil nematode abundance, taxonomic diversity, co-occurrence network [53]. Additionally, the observed pattern is based on 18S V4 region with primer 3NDf/1132rmod. While the 18S V4 primers used here are widely adopted, recent studies have highlighted that primer choice substantially influences nematode detection [74,75,76,77,78]. Certain taxa may therefore be underrepresented in our dataset. However, because our analyses focus on relative treatment comparisons, any amplification bias is expected to be consistent across samples and does not undermine our comparative conclusions. Root and litter quality are found to be critical factors in influencing nematode community composition and structure [50,68,79]. However, these data are not available, making it difficult to quantify their contribution. Future studies should incorporate seasonal sampling across multiple time points and directly measure litter chemical properties (e.g., C:N ratio, lignin content, secondary compounds) to disentangle the effects of forest type from those of litter chemistry and temporal dynamics. Furthermore, we acknowledge inherent limitations of the SFT approach in the present study. It cannot establish true causality or capture inter-annual variability and stochastic disturbances (e.g., drought, flood). Unmeasured confounding variables, such as historical land management practices or past stochastic events, may contribute to observed differences among current forest types. Therefore, our findings are best interpreted as spatial associations that suggest, rather than definitively confirm, succession trajectories. Long-term, repeated sampling would be required to fully disentangle temporal dynamics from spatial variation. Finally, interactions within soil biota communities, particularly nematode communities, are an integral part of ecological complexity, influencing community structure, stability, and resilience through the regulation of nutrient cycling and energy flow [40]. Our finding that network topology rather than diversity shaped nematode community responses to forest type is consistent with emerging views in soil microbiome research. Microbial community restructuring and interaction networks regulate ecosystem resilience and disease suppression [18], suggesting that network properties may be more functionally relevant than taxonomic diversity alone. Extending this reasoning to soil nematodes, our results imply that forest-type effects on co-occurrence network structure (e.g., reduced connectivity in coniferous stands) may have downstream consequences for soil food web stability and ecosystem functions such as decomposition and nutrient cycling. This aligns with the broader proposition that interaction networks, not just species inventories, are key determinants of ecosystem responses to environmental change. Given the known roles of nematode trophic groups in soil food webs, the observed shifts in community composition may have implications for nutrient cycling and ecosystem functioning. However, direct measurements of process rates (e.g., decomposition, nitrogen mineralization) would be required to test this hypothesis.

To deepen our understanding, future studies should focus on (1) disentangling the relative contributions of plant composition, soil properties, and management practices to guild trait variation using long-term paired experiments; (2) linking guild traits to ecosystem multifunctionality (e.g., carbon sequestration, nutrient retention, and pest resistance) rather than individual processes; and (3) exploring the role of microbial guild interactions (e.g., competition, mutualism) in mediating guild assembly in plantations. Such studies will inform evidence-based policies for balancing forest production and ecological conservation, particularly in the context of global afforestation initiatives and natural forest protection. This finding also highlights the conservation value of old-growth forests as biodiversity refuges.

To mitigate the functional limitations of monoculture plantations, management strategies could incorporate mixed-species planting and understory vegetation preservation to simulate the environmental heterogeneity of natural forests. For instance, introducing native tree species or retaining leaf litter diversity can promote the colonization of oligotrophic and symbiotic guilds, enhancing soil fertility and nutrient cycling complexity. Furthermore, monitoring guild traits (e.g., via functional annotation of microbial communities, stable isotope probing, or guild-specific marker gene sequencing) can serve as a bioindicator for assessing the ecological maturity of plantations, guiding adaptive management to align their functional traits with natural forest ecosystems.

## 5. Conclusions

Our results show that coniferous forests support significantly lower nematode trophic diversity than broad-leaved forests, indicating that forest types fundamentally shape soil food web structures. Distinct network structures and assembly processes are observed across these forest types. Mixed forests supported more complex, stable, and functionally integrated networks, whereas plantations formed dense but vulnerable networks. Ecologically, this suggests that conifer-driven reductions in nematode diversity may impair key soil functions, including organic matter decomposition and nutrient cycling, which are mediated by functionally diverse nematode communities. From a management perspective, our findings imply that maintaining mixed-species stands or broad-leaved corridors within conifer-dominated landscapes could help preserve soil biodiversity and ecosystem functioning. We recommend that forest managers consider nematode community composition as a potential bioindicator of soil health under different management regimes. This mechanistic insight that network stability and stochastic assembly prevail over simple taxonomic shifts provides a deeper understanding of how forest management impacts soil food web resilience and functioning. Recognizing these dynamics is essential for conserving soil health and ecosystem functions within forest environments. Future studies that integrate trait-based approaches, seasonal dynamics, and analyses of cross-kingdom interactions will enhance our understanding of nematode ecology and inform evidence-based forest management practices.

## Figures and Tables

**Table 1:** Dissimilarity analyses of soil nematode communities across forest types. Significant differences in overall community structure were tested using (1) permutational multivariate analysis of variance (PERMANOVA) based on Bray–Curtis distances, using the “Adonis2” function in the “vegan” package of R; (2) analysis of similarity (ANOSIM); and (3) multi-response permutation procedures (MRPP). Tests report the F/R statistic (PERMANOVA/ANOSIM) or the chance-corrected within-group agreement (A) for MRPP, degrees of freedom, and *p*-values (based on 999 permutations). All *p*-values were adjusted with the Benjamini–Hochberg (BH) method.

Object	Adonis			ANOSIM		MRPP		
	*F*	*R* ^2^	*Adjusted-p*	*R*	*Adjusted-p*	*A*	*δ*	*Adjusted-p*
Across Group	3.161	0.1224	0.001	0.327	0.001	0.044	0.754	0.001
Con vs. Dec	3.4256	0.0915	0.001	0.3954	0.001	0.0342	0.7608	0.001
Con vs. Mix	2.1603	0.0597	0.001	0.1380	0.001	0.0161	0.7416	0.001
Con vs. Pla	4. 5779	0.1187	0.001	0.5228	0.001	0.0474	0.7392	0.001
Dec vs. Mix	3. 1735	0.0854	0.001	0.3331	0.001	0.0312	0.7686	0.001
Dec vs. Pla	2.1573	0.0597	0.001	0.1968	0.001	0.0167	0.7677	0.001
Mix vs. Pla	3.5276	0.0940	0.001	0.3834	0.001	0.0350	0.7471	0.001

Note: Con, coniferous forest; Dec, broad-leaved forest; Mix, deciduous broad-leaved and evergreen broad-leaved mixed forest; Pla, plantation forest. All *p*-values were adjusted with the “Benjamini–Hochberg (BH)” method.

**Table 2:** Environmental vector fitting (EnvFit) results for soil variables and spatial predictors. Vectors show the strength (*r^2^*) and significance (*p*-value) of the correlation between each environmental/spatial variable and the major axes of variation in soil nematode community ordination (principal coordinate analysis (PCoA) based on Bray–Curtis dissimilarity). *p*-values were calculated using 999 permutations and adjusted for multiple comparisons using “Benjamini–Hochberg (BH)” method.

Variable	PCoA1	PCoA 2	r^2^	Pr(>r)
pH	−0.42204	−0.90658	0.6730	0.001
SOC	−0.21786	0.97598	0.3215	0.001
TN	−0.48324	0.87549	0.2120	0.001
TP	−0.79576	−0.60561	0.1551	0.004
SWC	0.22510	0.97434	0.3907	0.001
AN	−0.99988	0.01566	0.0988	0.020
AP	−0.99849	0.05500	0.1202	0.010
EC	−0.77323	−0.63412	0.5848	0.001
C/N	0.16059	0. 98702	0.2650	0.001
N/P	−0.09426	0.99555	0.1009	0.007
C/P	−0.09713	0.99527	0.1136	0.003
Longitude	−0.89281	−0.45403	0.4489	0.001
Latitude	0.78703	0.61691	0.5945	0.001
Altitude	0.69922	0.71491	0.6745	0.001

Note: AN, available nitrogen; AP, available phosphorus; C/N, ratio of soil organic carbon to total nitrogen; C/P, ratio of soil organic carbon to total phosphorus; N/P, ratio of total nitrogen to total phosphorus; SOC, soil organic carbon; SWC, soil water content; TN, total nitrogen; TP, total phosphorus.

## Data Availability

The original contributions presented in the study are included in the article, further inquiries can be directed to the corresponding authors.

## Supplementary Materials

The following supporting information can be downloaded at: https://www.mdpi.com/article/10.3390/microorganisms14051147/s1, Figure S1: Spearman correlations between soil nematode relative abundance/diversity and environmental variables. (A) Heatmap of Spearman correlation coefficients between the relative abundance of the top 20 nematode genera and 14 environmental variables. (B) Heatmap of correlation coefficients between five alpha diversity indices (observed OTUs, Faith PD, Shannon, Simpson, Pielou) and environmental variables. Color scale: blue = positive correlation, red = negative correlation. Significance correlations were indicated as follows: *, *p* < 0.05; **, 0.05 < *p* < 0.01, ***, *p* < 0.001. Non-significant correlations (*p* ≥ 0.05) are left unmarked. Environmental variables: pH, soil organic carbon (SOC), total nitrogen (TN), total phosphorus (TP), soil water content (SWC), available nitrogen (AN), available phosphorus (AP), electrical conductivity (EC), C/N ratio, N/P ratio, C/P ratio, longitude (Lon), latitude (Lat), altitude (Alt). n = 72 samples (4 forest types ×18 replicates); Figure S2: Topological attributes of soil nematode co-occurrence networks across forest types. Box plots comparing six network-level metrics among coniferous (Con), deciduous (Dec), mixed (Mix), and plantation (Pla) forests: (A) modularity, (B) clustering coefficient, (C) average path length, (D) edge density, (E) average degree, and (F) natural connectivity. Boxes represent IQR with median line; whiskers extend to 1.5×IQR; points are outliers. Statistical significance (Kruskal–Wallis test followed by Dunn’s post hoc with Benjamini–Hochberg correction) is indicated: *, *p* < 0.05; **, 0.05 < *p* < 0.01; ***, *p* < 0.001. Similar network construction parameters as in Figure 5. n = 1 network per forest type (derived from 18 samples each); Figure S3: Node-level centrality metrics of soil nematode co-occurrence networks across coniferous (Con), deciduous (Dec), mixed (Mix), and plantation (Pla) forests. Box plots of node attributes across forest types: (A) degree, (B) betweenness centrality, (C) closeness centrality, and (D) eigenvector centrality. Each point represents one OTU node. Only nodes present in the network (SparCC, |ρ| > 0.6, padj < 0.05) are shown. Nodes with zero connections are excluded. Significant differences among forest types (Kruskal–Wallis test) are indicated with asterisks as in Figure S2. The number of nodes per network: Con = 192, Dec = 177, Mix = 197, and Pla = 192.
