# Supplementary material for: Network Topology and Undominated Assembly Processes Govern Soil Nematode Community Responses to Forest Type
Source: Microorganisms. 2026 May 19;14(5):1147. doi: 10.3390/microorganisms14051147 (PMC13209612; doi:10.3390/microorganisms14051147)
Supplement: Supplementary file 1 [file microorganisms-14-01147-s001.zip › microorganisms-4248923-supplementary.pdf]

(**A**)

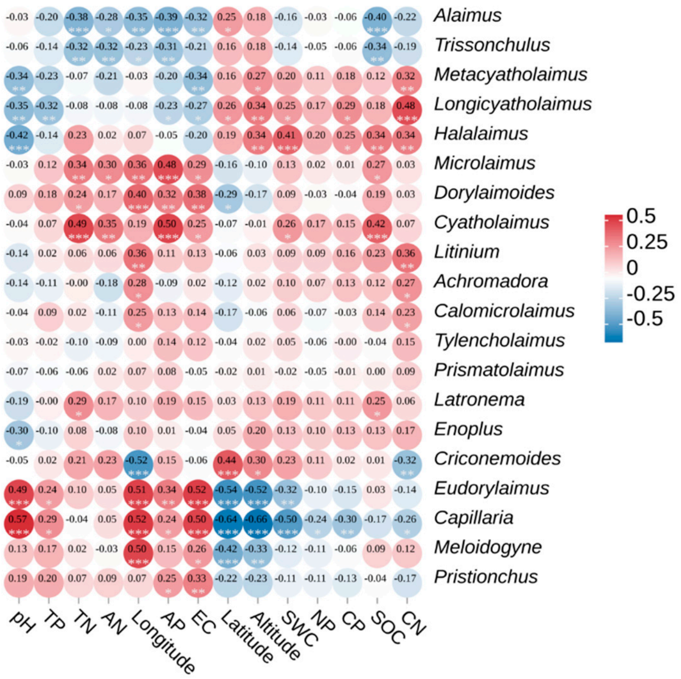

(**B**)

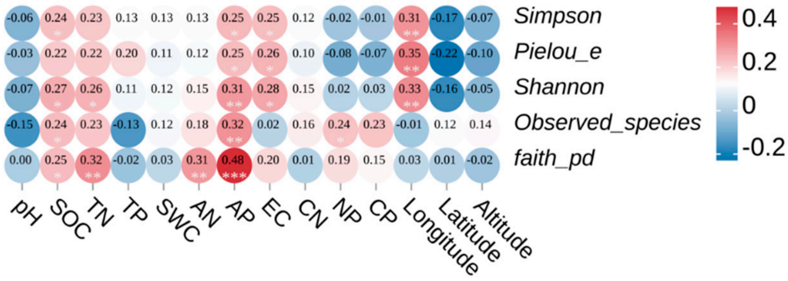

Figure S1. Spearman correlations between soil nematode relative abundance / diversity and environmental variables. (A) Heatmap of Spearman correlation coefficients between the relative abundance of the top 20 nematode genera and 14 environmental variables. (B) Heatmap of correlation coefficients between five alpha diversity indices (observed OTUs, Faith's PD, Shannon, Simpson, Pielou) and environmental variables. Color scale: blue = positive correlation, red = negative correlation. Significance correlations were indicated as follows: *, $p < 0.05$; **, $0.05 < p < 0.01$, and ***, $p < 0.001$. Non-significant correlations ($p \geq 0.05$) are left unmarked. Environmental variables: pH, soil organic carbon (SOC), total nitrogen (TN), total phosphorus (TP), soil water content (SWC), available nitrogen (AN), available phosphorus (AP), electrical conductivity (EC), C/N ratio, N/P ratio, C/P ratio,

longitude (Lon), latitude (Lat), altitude (Alt). n = 72 samples (4 forest types ×18 replicates).

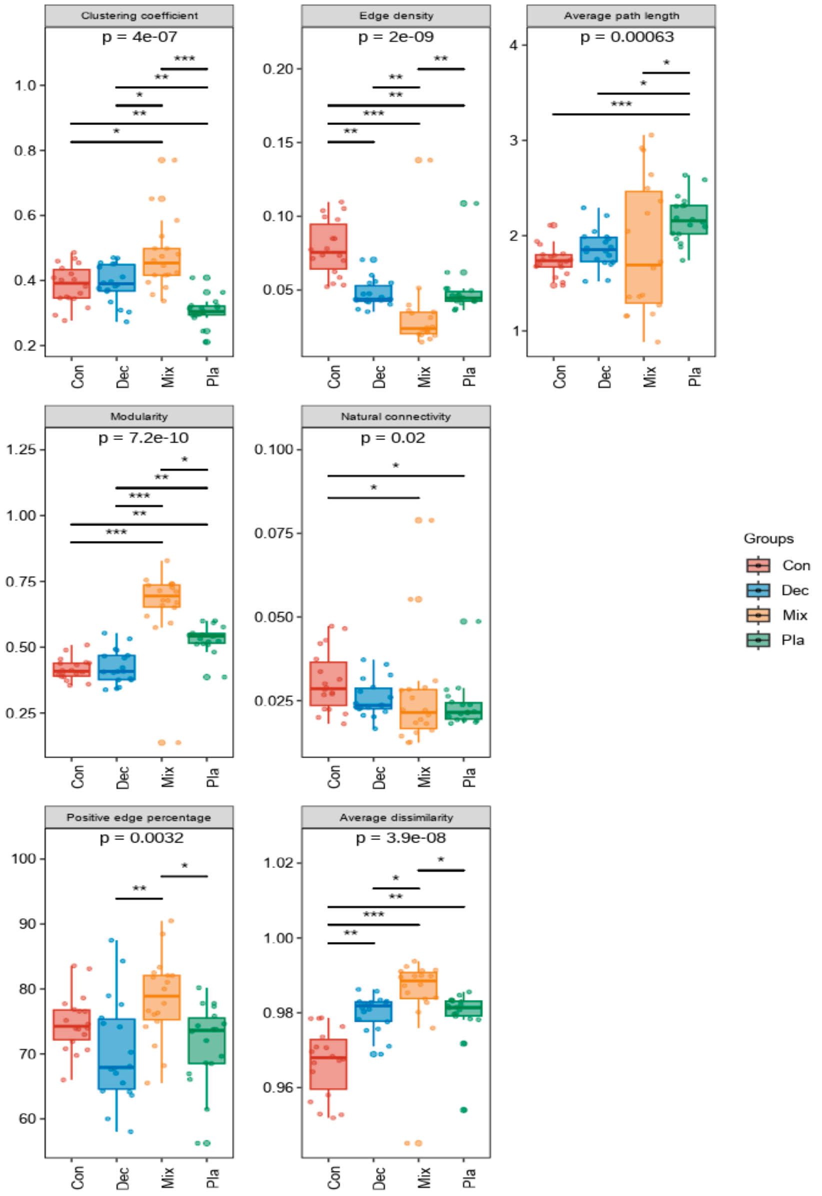

Figure S2. Topological attributes of soil nematode co-occurrence networks across forest types. Box plots comparing six network-level metrics among coniferous (Con), deciduous (Dec), mixed (Mix), and plantation (Pla) forests: (A) modularity, (B) clustering coefficient, (C) average path length, (D) edge density, (E) average degree, (F) natural connectivity. Boxes represent IQR with median line; whiskers extend to 1.5×IQR; points are outliers. Statistical significance (Kruskal–Wallis test followed by Dunn's post-hoc with Benjamini-Hochberg correction) is indicated: *, $p < 0.05$; **, 0.05

$< p < 0.01$; ***, $p < 0.001$. Network construction parameters as in Figure 5. n = 1 network per forest type (derived from 18 samples each)..

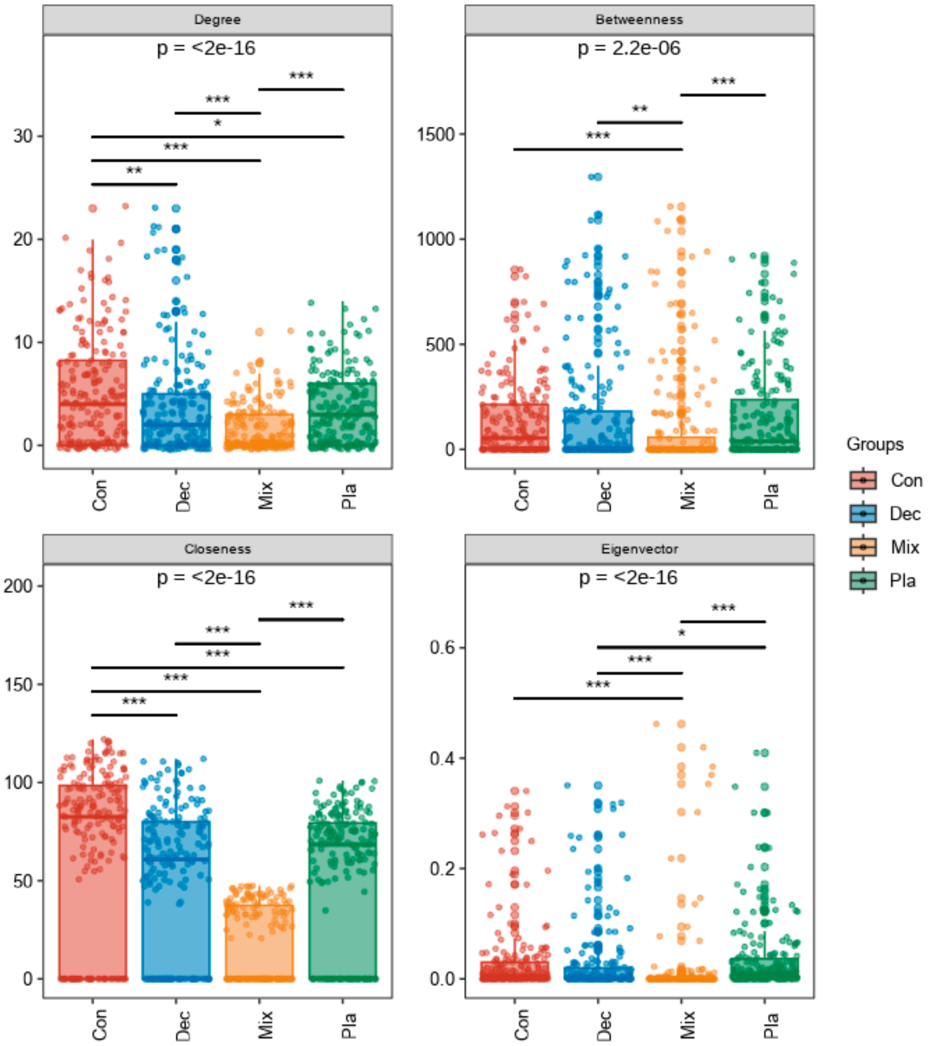

Figure S3. Node-level centrality metrics of soil nematode co-occurrence networks across coniferous (Con), deciduous (Dec), mixed (Mix), and plantation (Pla) forests. Box plots of node attributes across forest types: (A) degree, (B) betweenness centrality, (C) closeness centrality, (D) eigenvector centrality. Each point represents one OTU node. Only nodes present in the network (SparCC, $|\rho| > 0.6$, $p_{adj} < 0.05$) are shown. Nodes with zero connections are excluded. Significant differences among forest types (Kruskal–Wallis test) are indicated with asterisks as in Figure S2. The number of nodes per network: Con = 192, Dec = 177, Mix = 197, Pla = 192.
